# Ultrasensitive Label-free Electrochemical Immunosensor based on Multifunctionalized Graphene Nanocomposites for the Detection of Alpha Fetoprotein

**DOI:** 10.1038/srep42361

**Published:** 2017-02-10

**Authors:** Yaoguang Wang, Yong Zhang, Dan Wu, Hongmin Ma, Xuehui Pang, Dawei Fan, Qin Wei, Bin Du

**Affiliations:** 1 Key Laboratory of Chemical Sensing & Analysis in Universities of Shandong, School of Chemistry and Chemical Engineering, University of Jinan, Jinan 250022, P.R. China

## Abstract

In this work, a novel label-free electrochemical immunosensor was developed for the quantitative detection of alpha fetoprotein (AFP). Multifunctionalized graphene nanocomposites (TB-Au-Fe_3_O_4_-rGO) were applied to modify the electrode to achieve the amplification of electrochemical signal. TB-Au-Fe_3_O_4_-rGO includes the advantages of graphene, ferroferric oxide nanoparticles (Fe_3_O_4_ NPs), gold nanoparticles (Au NPs) and toluidine blue (TB). As a kind of redox probe, TB can produce the electrochemical signal. Graphene owns large specific surface area, high electrical conductivity and good adsorption property to load a large number of TB. Fe_3_O_4_ NPs have good electrocatalytic performance towards the redox of TB. Au NPs have good biocompatibility to capture the antibodies. Due to the good electrochemical performance of TB-Au-Fe_3_O_4_-rGO, the effective and sensitive detection of AFP was achieved by the designed electrochemical immunosensor. Under optimal conditions, the designed immunosensor exhibited a wide linear range from 1.0 × 10^−5^ ng/mL to 10.0 ng/mL with a low detection limit of 2.7 fg/mL for AFP. It also displayed good electrochemical performance including good reproducibility, selectivity and stability, which would provide potential applications in the clinical diagnosis of other tumor markers.

Alpha fetoprotein (AFP) is a tumor-associated fetal protein produced by the fetal liver and yolk sac^1^. The expressional level of AFP is highly elevated in hepatocellular carcinoma^2^,^3^. Therefore, AFP has been considered as one of the most important tumor markers in diagnosing and targeting of hepatocellular carcinoma^4^. A number of methods have been proposed for the quantitative detection of AFP, such as electrochemical immunoassay^5^, photoelectrochemical immunoassay^6^, fluoresencent immunoassay^7^, chemiluminescent immunoassay^8^ and enzyme-linked immunosorbent assay (ELISA)^9^, etc. Compared with other methods, the electrochemical immunosensor has many advantages such as economical, sensitive, portable and simple-to-operate^10^,^11^,^12^,^13^.

As a star among two-dimensional nanomaterials, graphene has attracted tremendous research interest in the field of electrochemistry due to their intrinsic properties, including the electronic, optical, and mechanical properties associated with their planar structure^14^,^15^. In general, the large specific surface area and the high electrical conductivity are the two key factors to promote the application of graphene in electrochemical immunosensors. For example, Tian *et al*. have developed chemically functionalized Ag/Au nanoparticles coated on graphene for the detection of carcinoembryonic antigen (CEA) in clinical immunoassay^16^. And Thavarungkul *et al*. have developed and tested a novel highly sensitive electrochemical immunosensor based on Au nanoparticles-graphene-chitosan nanocomposites^17^. Therefore, graphene-based electrochemical immunosensor can achieve a high sensitivity and a good stability^16^,^17^,^18^. In addition, graphene has another advantage of the good adsorption property^19^,^20^, which is seldom developed and used in electrochemical immunosensors.

According to our previous reports^21^,^22^,^23^,^24^, reduced graphene oxide functionalized with ferroferric oxide (Fe_3_O_4_-rGO) can be used in adsorbing a variety of heavy metals. In this case, a label-free electrochemical immunosensor based on multifunctionalized graphene nanocomposites (TB-Au-Fe_3_O_4_-rGO) was designed for the quantitative detection of AFP. First of all, gold nanoparticles (Au NPs) which could immobilize the antibodies through the good biocompatibility were applied to functionalize the Fe_3_O_4_-rGO to obtain the Au-Fe_3_O_4_-rGO^25^,^26^,^27^,^28^,^29^,^30^,^31^,^32^,^33^,^34^,^35^. Subsequently, taking advantage of the good adsorption property, Au-Fe_3_O_4_-rGO was used to adsorb toluidine blue (TB) to obtain the TB-Au-Fe_3_O_4_-rGO. TB is a kind of organic dye and widely used as an electron transfer mediator for providing electrochemical signal in the electrochemical immunosensor^36^. As is known to all, ferroferric oxide nanoparticles (Fe_3_O_4_ NPs) have a good electrocatalytic performance^37^,^38^,^39^,^40^, which can promote the redox of TB. In addition, the graphene can provide large specific surface area for the immobilization of antibodies and high electrical conductivity for the electron transfer. Therefore, the usage of the TB-Au-Fe_3_O_4_-rGO modified electrode to fabricate the electrochemical immunosensor can achieve the effective and sensitive detection of AFP.

## Experimental

### Apparatus and reagents

All electrochemical measurements were performed on a CHI760E electrochemical workstation (Huakeputian Technology Beijing Co., Ltd., China). A conventional three-electrode system was used for all electrochemical measurements: a glassy carbon electrode (GCE, 4 mm in diameter) as the working electrode, a saturated calomel electrode (SCE) as the reference electrode, and a platinum wire electrode as the counter electrode. Scanning electron microscope (SEM) images were obtained by using Quanta FEG250 field emission environmental SEM (FEI, United States) operated at 4 KV.

Human AFP and antibody to human AFP (anti-AFP) were purchased from Beijing Dingguo Changsheng Biotechnology Co., Ltd., China. Bovine serum albumin (BSA) and TB were purchased from Shanghai Sinopharm Chemical Reagent Co., Ltd., China. Sodium acetate trihydrate (NaAc·3H_2_O), chloroauric acid (HAuCl_4_·4H_2_O) and trisodium citrate were purchased from Shanghai Aladdin Chemistry Co., Ltd, China. Ethanol, ethylene glycol (EG) and ethanediamine (EDA) were purchased from Tianjin Fuyu Fine Chemical Co., Ltd., China. Ferric chloride hexahydrate (FeCl_3_·6H_2_O) was purchased from Tianjin Damao Chemical Reagent Co., Ltd., China. Phosphate buffered saline (PBS 1/15 M Na_2_HPO_4_ and KH_2_PO_4_) was used as an electrolyte for all electrochemical measurements, which was purged with nitrogen gas for 20 min to remove the dissolved oxygen. All other reagents were of analytical grade and ultrapure water was used throughout the study.

### Synthesis of the TB-Au-Fe_3_O_4_-rGO

**Figure 1:**
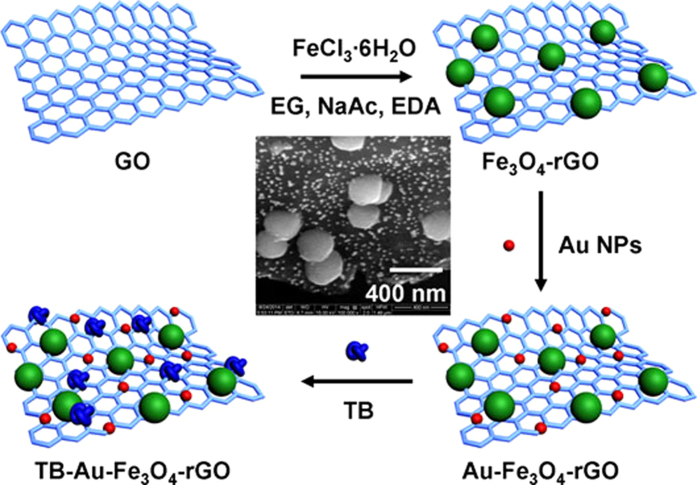
The synthesis procedure of the TB-Au-Fe_3_O_4_-rGO and SEM image of the Au-Fe_3_O_4_-rGO (inset).

Figure 1 shows the synthesis procedure of the TB-Au-Fe_3_O_4_-rGO. SEM image was performed to characterize the shape and size of the Au-Fe_3_O_4_-rGO (inset of Fig. 1). Graphene oxide (GO) was synthesized by an improved Hummers method^41^. In brief, a mixture of concentrated H_2_SO_4_ (36 mL) and H_3_PO_4_ (4 mL) was added into a mixture of graphite flakes (0.3 g) and KMnO_4_ (1.8 g), producing a slight exotherm to 35~40 °C. The reaction was then heated to 50 °C and stirred for 12 h. After that, the reaction was cooled to room temperature and poured onto ice (40 mL) with 30% H_2_O_2_ (0.3 mL), and the mixture was centrifuged and the supernatant was decanted away. For workup, the remaining solid material was washed in succession with water, 0.2 M HCl, ethanol and ether. The obtained solid was dried in vacuum overnight.

In a typical synthesis of Fe_3_O_4_-rGO^21^. FeCl_3_·6H_2_O (0.5 g) was dissolved in EG (10 mL) to form a clear solution, followed by the addition of NaAc (1.5 g), EDA (5 mL) and GO (0.5 g). The mixture was stirred vigorously for 30 min and then sealed in a teflon-lined stainless steel autoclave. The autoclave was heated to 200 °C and maintained for 8 h, and then was cooled to room temperature. The resulting black powder was washed several times and dried at 35 °C under high vacuum overnight. It should be noted that the GO was translated into the reduced graphene oxide (rGO) in the process of reaction.

Au NPs were synthesized by the classical Frens method^42^. In brief, a solution of HAuCl_4_ (0.01 wt%, 100 mL) was heated to boiling, and then a solution of trisodium citrate (1 wt%, 1.5 mL) was added. The boiling solution turned a brilliant ruby-red in around 15 min, indicating the formation of Au NPs, and then it was cooled to room temperature.

A mixture of Fe_3_O_4_-rGO (20 mg) and the prepared Au NPs solution (40 mL) was shaked for 12 h, The resulting Au-Fe_3_O_4_-rGO was obtained by washed several times and dried at 35 °C under high vacuum overnight. 1 mL of 2 mg/mL Au-Fe_3_O_4_-rGO aqueous solution was mixed with 1 mL of 2 mg/mL TB solution under stirring for another 12 h. The final product was washed thoroughly and redispersed in 1 mL of water.

### Fabrication of the immunosensor

**Figure 2:**
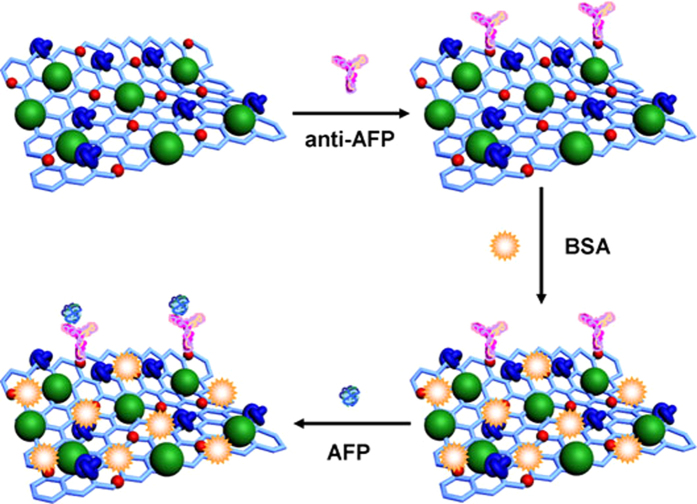
The schematic diagram of the label-free electrochemical immunosensor fabricated on the GCE.

Figure 2 shows the schematic diagram of the label-free electrochemical immunosensor fabricated on the GCE. A GCE was polished to a mirror-like finish with alumina powder (1.0, 0.3 and 0.05 μm), and then it was thoroughly cleaned before use. First, an aqueous solution of TB-Au-Fe_3_O_4_-rGO (2 mg/mL, 6 μL) was added onto the surface of bare GCE and then dried. After washed, anti-AFP dispersion (10 μg/mL, 6 μL) was added onto the electrode. According to the ref. 43, anti-AFP can be conjugated on the surface of TB-Au-Fe_3_O_4_-rGO due to the bonding between amino groups of antibodies and Au NPs. After incubated at 4 °C for 1 h and washed, BSA solution (10 mg/mL, 3 μL) was added onto the electrode to eliminate nonspecific binding sites. After incubated for another 1 h at 4 °C, the electrode was washed and incubated with a varying concentration of AFP (1.0 × 10^−5^ ~ 10.0 ng/mL, 6 μL) for 1 h at room temperature, and then the electrode was washed extensively to remove unbounded AFP molecules for measurement. For Square Wave Voltammetry (SWV) to record the amperometric response in PBS at pH = 6.8, a detection potential from −0.6 V to 0 V was selected. The response time of one electrode is 30 seconds.

## Results and Discussion

### SEM and EDX characterization

**Figure 3:**
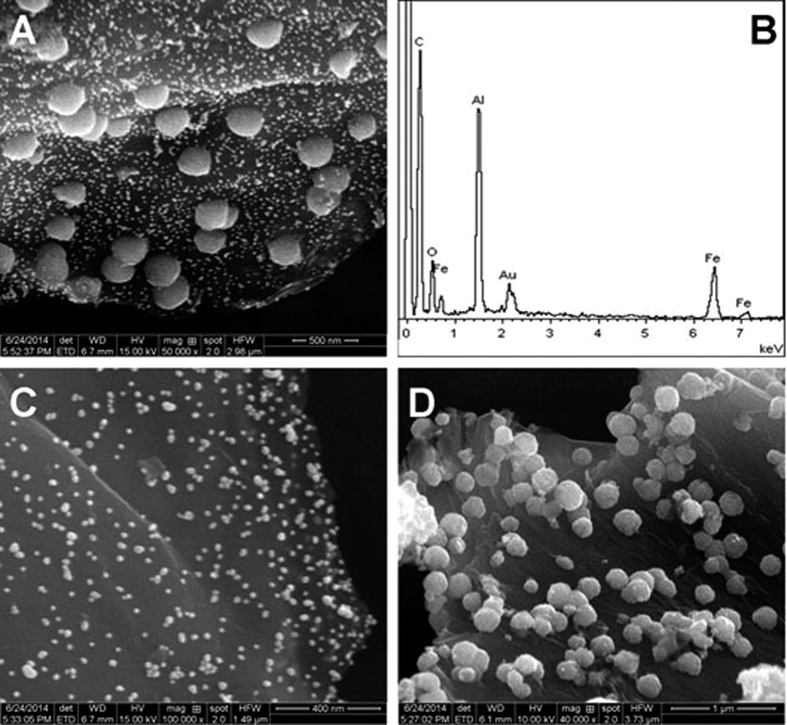
SEM images of Au-Fe_3_O_4_-rGO (**A**), Au-rGO (**C**) and Fe_3_O_4_-rGO (**D**); EDX spectrum of Au-Fe_3_O_4_-rGO (**B**).

Figure 3A shows SEM image of Au-Fe_3_O_4_-rGO. It can be observed that the rGO of Au-Fe_3_O_4_-rGO is thin and wrinkled, which resembles crumpled silk veil waves. The Fe_3_O_4_ NPs are loaded on the surface of rGO with an average diameter about 200 nm. A large number of Au NPs with an average size about 20 nm are successfully loaded on the surface of Fe_3_O_4_-rGO. EDX spectrum of Au-Fe_3_O_4_-rGO (Fig. 3B) was used to further explain what kinds of elements were contained in Au-Fe_3_O_4_-rGO. Al element can be observed because the sample was transferred on a clean aluminium foil for EDX analysis. Au-rGO and Fe_3_O_4_-rGO were also synthesized for the control experiments^44^. The Au-rGO retains the thin and wrinkled structure of rGO, which is loaded with massive Au NPs (Fig. 3C). In the SEM image of Fe_3_O_4_-rGO (Fig. 3D), the rGO presents lamellar fold structure and numbers of Fe_3_O_4_ NPs with a uniform diameter of about 200 nm are loaded on the surface of it.

### Optimization of experimental conditions

**Figure 4:**
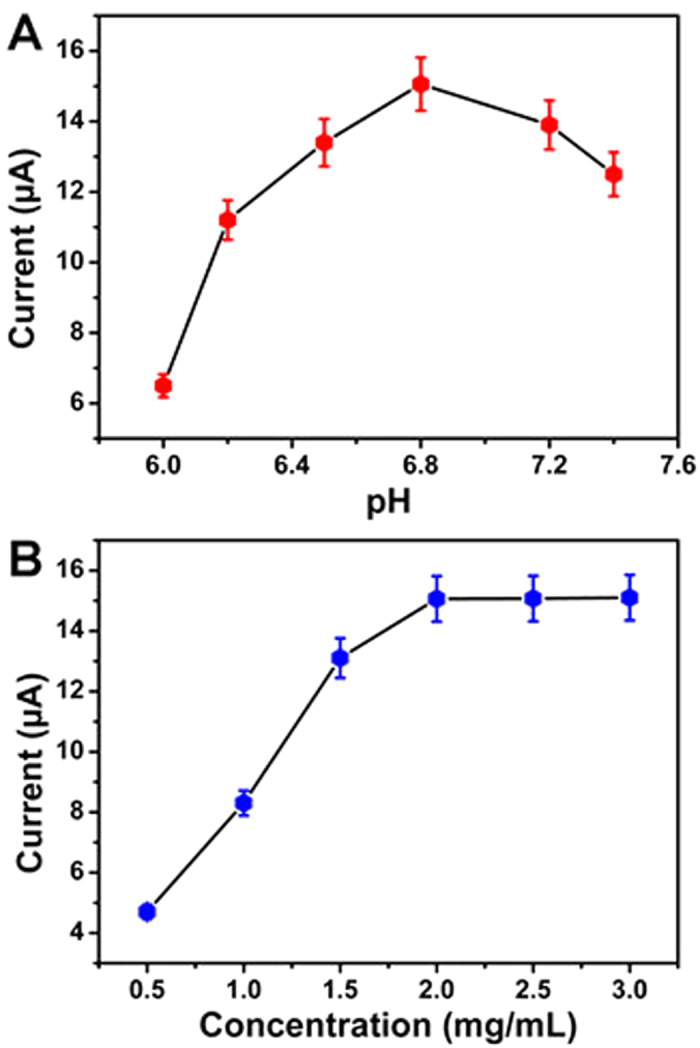
Effect of pH (**A**) and the concentration of TB-Au-Fe_3_O_4_-rGO (**B**) on the electrochemical current responses of the immunosensor for the detection of 10.0 ng/mL of AFP. Error bar = RSD (*n* = 5).

In order to achieve an optimal electrochemical signal, optimizations of experimental conditions are necessary. The pH value of PBS and the concentration of TB-Au-Fe_3_O_4_-rGO influence the electrochemical current response. Figure 4A shows the different electrochemical current responses of the electrode modified with 2 mg/mL TB-Au-Fe_3_O_4_-rGO in different pH values of PBS for the detection of 10.0 ng/mL of AFP. Figure 4B shows the different electrochemical current responses of the electrode modified with different concentrations of TB-Au-Fe_3_O_4_-rGO in PBS at pH = 6.8 for the detection of 10.0 ng/mL of AFP. As shown in these figures, the optimal amperometric response was achieved at a pH of 6.8 and at a concentration of 2 mg/mL. Therefore, PBS at pH = 6.8 and 2 mg/mL TB-Au-Fe_3_O_4_-rGO were selected for the test throughout this study.

In addition, the other factors were controlled strictly, for example, the incubation time was 1 h, the incubation temperature was the room temperature, and the concentration of antibodies was 10 μg/mL. All these above factors would make sure antibodies and antigens could effectively and specifically recognize with each other.

### Electrochemical characterization

**Figure 5:**
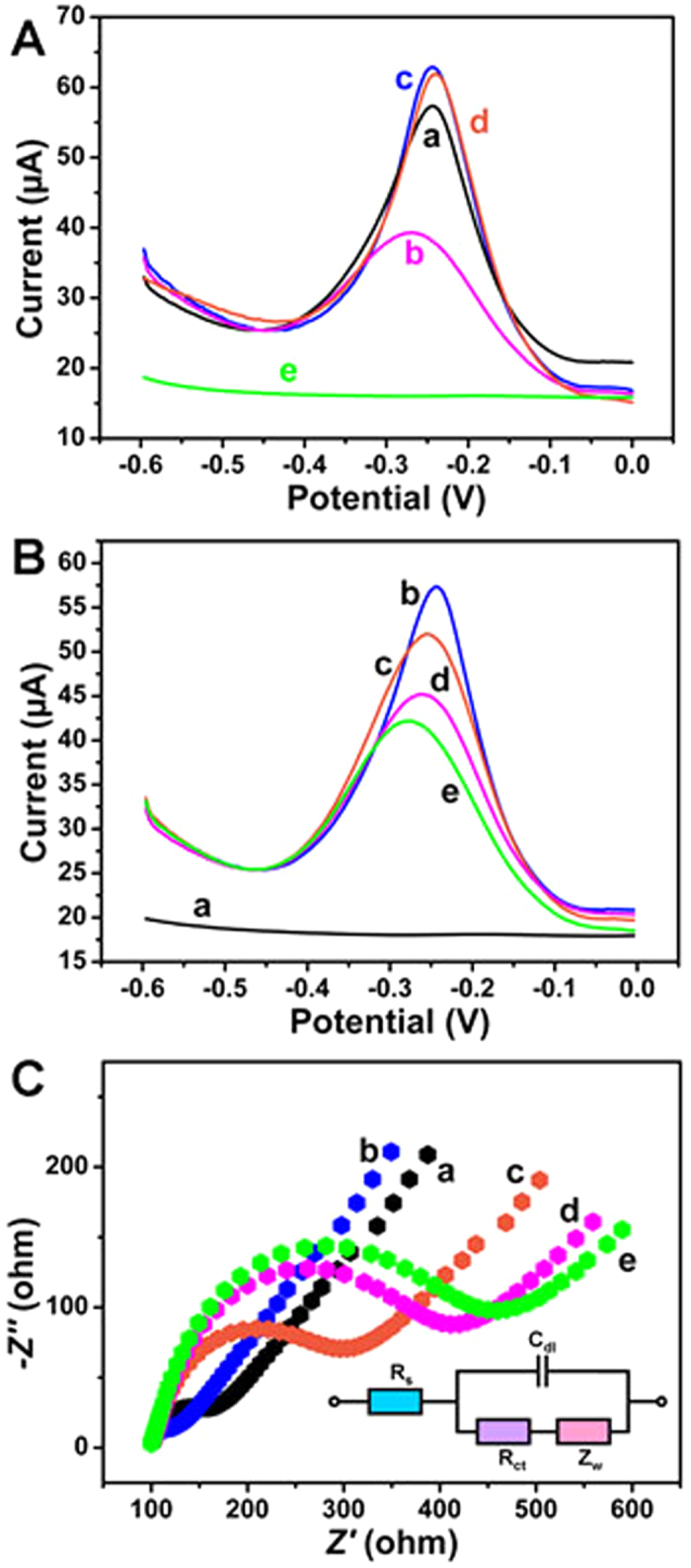
Electrochemical current responses recorded from −0.6 V to 0 V in PBS at pH 6.8 (**A**): TB-Au-Fe_3_O_4_-rGO/GCE (a), TB-Au-rGO/GCE (b), TB-Fe_3_O_4_-rGO/GCE (c), anti-AFP/TB-Fe_3_O_4_-rGO/GCE (d) and Au-Fe_3_O_4_-rGO/GCE (e); Electrochemical current responses recorded from −0.6 V to 0 V in PBS at pH 6.8 (**B**) and Nyquist plots of the A.C. impedance method (**C**): bare GCE (a), TB-Au-Fe_3_O_4_-rGO/GCE (b), anti-AFP/TB-Au-Fe_3_O_4_-rGO/GCE (c), BSA/anti-AFP/TB-Au-Fe_3_O_4_-rGO/GCE (d) and AFP/BSA/anti-AFP/TB-Au-Fe_3_O_4_-rGO/GCE (e); Inset shows the Randles model for the equivalent circuit, which represents each component at the working electrode interface and in the solution during the electrochemical reaction in the presence of Fe(CN)_6_^3−^/Fe(CN)_6_^4−^: solution resistance (R_s_), electron transfer resistance (R_ct_), capacitance of double layer (C_dl_), Warburg impedance (Z_w_).

In order to investigate the role of TB, Fe_3_O_4_ NPs and Au NPs in the fabrication of the label-free electrochemical immunosensor, SWV curves of TB-Au-Fe_3_O_4_-rGO/GCE (curve a), TB-Au-rGO/GCE (curve b), TB-Fe_3_O_4_-rGO/GCE (curve c), anti-AFP/TB-Fe_3_O_4_-rGO/GCE (curve d) and Au-Fe_3_O_4_-rGO/GCE (e) were recorded in PBS at pH 6.8. As shown in Fig. 5A, Au-Fe_3_O_4_-rGO/GCE modified electrode has no obvious electrochemical current response (curve e). However, TB-Au-Fe_3_O_4_-rGO modified electrode (curve a) has an obvious electrochemical current response. It indicates that TB can produce the electrochemical current response for the immunosensor. In addition, TB-Au-rGO modified electrode (curve b) has an electrochemical current response about the half of TB-Au-Fe_3_O_4_-rGO modified electrode. It indicates that Fe_3_O_4_ NPs have a good electrocatalytical performance to promote the redox of TB. TB-Fe_3_O_4_-rGO modified electrode (curve c) has a slightly larger electrochemical current response than TB-Au-Fe_3_O_4_-rGO modified electrode. It indicates that Fe_3_O_4_-rGO can load more TB than Au-Fe_3_O_4_-rGO and the increase of the surface area cannot lead to the increase of electrochemical current response. But after modified with anti-AFP (curve d), the electrochemical current response has no obvious change. It indicates that anti-AFP cannot be captured on the surface of TB-Fe_3_O_4_-rGO without Au NPs. Therefore, Au NPs with a good biocompatibility can capture the antibodies.

SWV was also used to verify the successful fabrication of the immunosensor (Fig. 5B). It can be observed that the bare GCE (curve a) modified with TB exhibits an obvious electrochemical peak current at −0.24 V (curve b). After modified with anti-AFP (curve c), BSA (curve d) and AFP (curve e), the decreasing electrochemical current response indicates biological active substances can hinder the efficiency of electron transfer. The slight shift of the electrochemical peak potential might be due to the modification of the biological active substance.

In order to further characterize the fabrication process of the label-free electrochemical immunosensor, Nyquist plots of the A.C. impedance method was recorded from 1 to 10^5^ Hz at 0.2 V in a solution containing 0.1 M KCl and 2.5 mM Fe(CN)_6_^3−^/Fe(CN)_6_^4−^. Nyquist plots are consisted of two portions. The linear portion at low frequencies is associated with electrochemical behavior limited by diffusion. The semicircle portion at high frequencies is associated with the electrochemical process subject to electron transfer, where the diameter corresponds to the resistance. Simply, resistance change could be judged by observing the diameter change of semicircle portion. Thus, A.C. impedance is a suitable method for monitoring the changes in the surface features during the fabrication process^45^,^46^,^47^,^48^. The inset in Fig. 5C shows the Randles model for the equivalent circuit^49^,^50^,^51^, which represents each component at the working electrode interface and in the solution during the electrochemical reaction in the presence of Fe(CN)_6_^3−^/Fe(CN)_6_^4−^: solution resistance (R_s_), charge transfer resistance (R_ct_), capacitance of double layer (C_dl_), Warburg impedance (Z_w_). As shown in the Fig. 5C, it can be observed that the bare GCE exhibits a very small resistance (curve a), which is characteristic of a diffusion-limiting step in the electrochemical process. After the modification of TB-Au-Fe_3_O_4_-rGO, the electrode shows a decreasing resistance (curve b). It implies that the successful immobilization of TB-Au-Fe_3_O_4_-rGO on the surface of bare GCE and TB-Au-Fe_3_O_4_-rGO can provide a sensitive interface to accelerate the electron transfer. The gradually increasing resistance of electrodes further modified with anti-AFP (curve c) and BSA (curve d) indicates the successful immobilization of the non-conductive bioactive substances. It also indicates that TB-Au-Fe_3_O_4_-rGO can offer a biocompatible surface for the capture of anti-AFP. After the modification of AFP, the electrode shows an increasing resistance (curve e), implying that the successful specific recognition between anti-AFP and AFP.

### Characterization of the immunosensor

**Figure 6:**
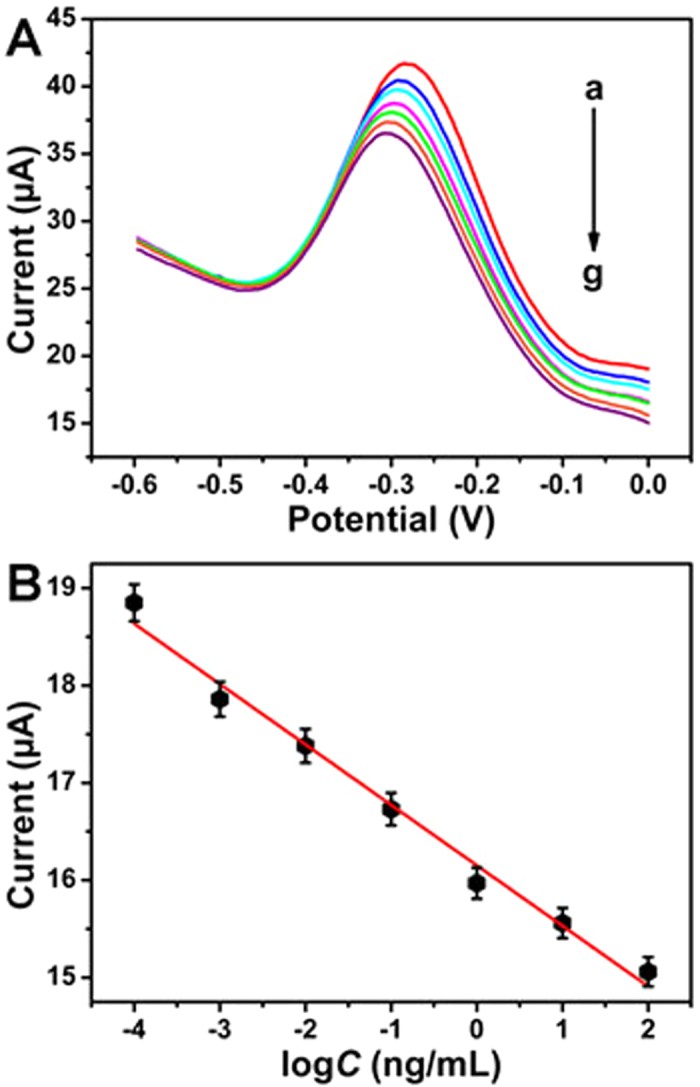
(**A**) Electrocatalytic current responses of the immunosensor for the detection of different concentrations of AFP: 1.0 × 10^−5^ ng/mL (a), 1.0 × 10^−4^ ng/mL (b), 1.0 × 10^−3^ ng/mL (c), 1.0 × 10^−2^ ng/mL (d), 0.1 ng/mL (e), 1.0 ng/mL (f) and 10.0 ng/mL (g); (**B**) Calibration curve of the immunosensor for the detection of different concentrations of AFP. Error bar = RSD (*n* = 5).

Under optimal conditions, the label-free electrochemical immunosensor was employed to detect different concentrations of AFP. Figure 6A shows the electrocatalytic current responses of the designed immunosensor for the detection of AFP covering the concentration range from 1.0 × 10^−5^ ng/mL (curve a) to 10.0 ng/mL (curve g). Figure 6B shows a linear relationship between electrocatalytic current responses and the logarithmic values of AFP concentration. The electrocatalytic current responses have a linear relationship with the logarithmic values of AFP concentration. And the linear regression equation of the calibration curve was *I* = −0.62 log*C* + 16.15 with correlation coefficient of 0.99. According to the refs 52 and 53, the detection limit can be calculated with the equation of *c*_L_ = 10^(*I*−16.15)/–0.62^, where *c*_L_ is the detection limit and *I* is given by the equation of *x*_b1_* + 3s*_b1_ (*x*_b1_ is the mean signal of blank measures, *s*_b1_ is the standard deviation of five parallel blank measures, the *x*_b1_ = 19.50 and *s*_b1_ = 0.034 in this work). The low detection limit of 2.7 fg/mL was obtained, which was ascribed to the novel signal amplification strategy based on multifunctionalized graphene nanocomposites in the fabrication of the designed immunosensor. The linear range and detection limit of the proposed immunosensor were compared with other reported methods for the detection of AFP in Table S1. It could be found that the proposed immunosensor showed a satisfactory linear range and detection limit.

### Reproducibility, selectivity and stability

**Figure 7:**
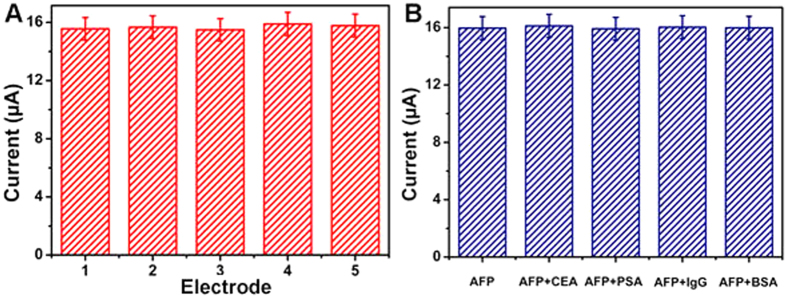
(**A**) Electrochemical signal responses of the immunosensor fabricated on five different electrodes for the detection of 1.0 ng/mL AFP; (**B**) Electrochemical signal responses of the immunosensor to 1.0 ng/mL AFP, 1.0 ng/mL AFP + 100 ng/mL CEA, 1.0 ng/mL AFP + 100.0 ng/mL PSA, 1.0 ng/mL AFP + 100.0 ng/mL IgG and 1.0 ng/mL AFP + 100.0 ng/mL BSA. Error bar = RSD (*n* = 5).

To evaluate the reproducibility of the label-free electrochemical immunosensor, a series of five electrodes were prepared for the detection of 1.0 ng/mL AFP (Fig. 7A). The relative standard deviation (RSD) of the measurements for the five electrodes was less than 5%, suggesting the precision and reproducibility of the designed immunosensor was quite good.

To investigate the specificity of the label-free electrochemical immunosensor, interference study was performed by using CEA, prostate specific antigen (PSA), human immunoglobulin (IgG) and BSA. 1.0 ng/mL AFP with and without 100.0 ng/mL interfering substances solution was measured by the designed immunosensor (Fig. 7B). It can be observed that the electrochemical current response variation due to the interfering substances was less than 5% of that without interferences, indicating the selectivity of the designed immunosensor was excellent.

To test the stability of the fabricated electrochemical immunosensor, it was stored at 4 °C when not in use. After one month, the electrochemical current response change was less than 5% for the detection of 1.0 ng/mL AFP. The good stability of the designed immunosensor can be ascribed to the good biocompatibility of the multifunctionalized graphene nanocomposites. The reproducibility, selectivity and stability of the designed immunosensor were all acceptable, thus it was suitable for quantitative detection of AFP in real human samples.

### Real sample analysis

In order to validate the label-free electrochemical immunosensor, a comparative experiment with the commercialized available ELISA method for the detection of AFP in human serum sample was conducted (Table S2). The relative error between the two methods was in the range from −4.9% to 4.0%. These data revealed a good agreement between the two analytical methods, indicating the feasibility of the designed immunosensor for clinical application.

## Conclusion

A novel label-free electrochemical immunosensor for the quantitative detection of AFP has been developed in this work. Thanks to the good adsorption property and the good electrochemical performance of the multifunctionalized graphene nanocomposites, the proposed immunosensor showed a wide linear range, a low detection limit, good reproducibility, fine selectivity and acceptable stability. The designed immunosensor might provide wide potential applications for the detection of AFP in clinical diagnosis and also can be applied in clinical detection of other tumor markers.

## Additional Information

**How to cite this article:** Wang, Y. *et al*. Ultrasensitive Label-free Electrochemical Immunosensor based on Multifunctionalized Graphene Nanocomposites for the Detection of Alpha Fetoprotein. *Sci. Rep.* **7**, 42361; doi: 10.1038/srep42361 (2017).

**Publisher's note:** Springer Nature remains neutral with regard to jurisdictional claims in published maps and institutional affiliations.

## Supplementary Material

Supplementary Materials
