# Supplementary material for: Ultrasensitive Label-free Electrochemical Immunosensor based on Multifunctionalized Graphene Nanocomposites for the Detection of Alpha Fetoprotein
Source: Sci Rep. 2017 Feb 10;7:42361. doi: 10.1038/srep42361 (PMC5301246; doi:10.1038/srep42361)
Supplement: Supplementary Materials [file srep42361-s1.pdf]

Supplementary materials

Ultrasensitive label-free electrochemical immunosensor based on multifunctionalized graphene nanocomposites for the detection of alpha fetoprotein

Yaoguang Wang, Yong Zhang, Dan Wu, Hongmin Ma, Xuehui Pang, Dawei Fan, Qin Wei, Bin Du*

Key Laboratory of Chemical Sensing & Analysis in Universities of Shandong, School of Chemistry and Chemical Engineering, University of Jinan, Jinan 250022, P.R. China.

*Corresponding author. Tel. + 86 531 82767872; fax: + 86 531 82767367.

E-mail address: dubin61@gmail.com

Table S1 Comparison of different methods for the detection of AFP

Method	Linear range	Detection limit	Reference
Label-free electrochemiluminescence immunosensor	0.0005 ~ 50 pg/mL	0.2 fg/mL	1
Sandwich-type electrochemiluminescence immunosensor	0.005 ~ 0.2 pg/mL	1.0 fg/mL	2
Label-free electrochemical immunosensor	0.00005 ~ 250 ng/mL	1.1 fg/mL	3
Sandwich-type electrochemical immunosensor	0.0001 ~ 50 ng/mL	33 fg/mL	4
Sandwich-type electrochemical immunosensor	0.0001 ~ 10 ng/mL	0.05 pg/mL	5
Label-free electrochemical impedance spectroscopy immunosensor	1 ~ 100 pg/mL	0.1 pg/mL	6
Label-free electrochemical immunosensor	0.001 ~ 45 ng/mL	0.13 pg/mL	7
Sandwich-type electrochemical immunosensor	0.001 ~ 5 ng/mL	0.2 pg/mL	8
Sandwich-type electrochemical immunosensor	0.01 ~ 60 ng/mL	1.6 pg/mL	9
Label-free electrochemiluminescence immunosensor	0.005 ~ 14 ng/mL	2.0 pg/mL	10
Sandwich-type electrochemical immunosensor	0.01 ~ 40 ng/mL	2.3 pg/mL	11
Label-free electrochemiluminescent immunosensor	0.01 ~ 100 ng/mL	3.3 pg/mL	12
Label-free electrochemical immunosensor	0.01 ~ 75 ng/mL	4.0 pg/mL	13
Label-free electrochemical immunosensor	0.01 ~ 12 ng/mL	5.0 pg/mL	14
Sandwich-type electrochemical immunosensor	0.05 ~ 30 ng/mL	5.0 pg/mL	15
Competitive electrochemical immunosensor	0.05 ~100 ng/mL	5.0 pg/mL	16
Label-free photoelectrochemical immunosensor	0.1 ~ 500 ng mL	10 pg/mL	17
Label-free electrochemical immunosensor	0.1 ~ 30 ng mL	18 pg/mL	18
Sandwich-type electrochemiluminescence immunosensor	0.05 ~ 50 ng/mL	30 pg/mL	19
Sandwich-type electrochemical immunosensor	0.5 ~ 80 ng/mL	250 pg/mL	20
Label-free electrochemical immunosensor	0.00001 ~10.0 ng/mL	2.7 fg/mL	This method

Table S2 Human serum sample analysis using the designed method and the ELISA method

Sample	This method (ng/mL) [a]	ELISA (ng/mL) [a]	Relative error (%)
1	2.22	2.14	3.7
2	4.42	4.65	-4.9
3	7.52	7.23	4.0

[a] Each value is the average of five measurements.

References:

1. Guo, Z., Hao, T., Duan, J., Wang, S. & Wei, D. Electrochemiluminescence immunosensor based on graphene-CdS quantum dots-agarose composite for the ultrasensitive detection of alpha fetoprotein. *Talanta* **89**, 27-32 (2012).
2. Wang, H., Sun, D., Tan, Z., Gong, W. & Wang, L. Electrochemiluminescence immunosensor for α-fetoprotein using $Ru(bpy)_3^{2+}$-encapsulated liposome as labels. *Colloid. Surface. B* **84**, 515-519 (2011).
3. Liu, K., Zhang, J., Liu, Q. & Huang, H. Electrochemical immunosensor for alpha-fetoprotein determination based on ZnSe quantum dots/Azure I/gold nanoparticles/poly (3,4-ethylenedioxythiophene) modified Pt electrode. *Electrochim. Acta* **114**, 448-454 (2013).
4. Wei, Y. et al. Sandwich-type electrochemical immunosensor for the detection of AFP based on Pd octahedral and APTES-M-CeO_2-GS as signal labels. *Biosens. Bioelectron.* **79**, 482-487 (2016).
5. Jiao, L. et al. Graphene loaded bimetallic Au@Pt nanodendrites enhancing ultrasensitive electrochemical immunoassay of AFP. *Sensor. Actuat. B-Chem.* **231**, 513-519 (2016).
6. Yang, H. et al. Detection and discrimination of alpha-fetoprotein with a label-free electrochemical impedance spectroscopy biosensor array based on lectin functionalized carbon nanotubes. *Talanta* **111**, 62-68 (2013).
7. Kavosi, B., Hallaj, R., Teymourian, H. & Salimi, A. Au nanoparticles/PAMAM dendrimer functionalized wired ethyleneamine-viologen as highly efficient interface

for ultra-sensitive α-fetoprotein electrochemical immunosensor. *Biosens. Bioelectron.* **59**, 389-396 (2014).

8. Gao, J. et al. A novel electrochemical immunosensor using β-cyclodextrins functionalized silver supported adamantine-modified glucose oxidase as labels for ultrasensitive detection of alpha-fetoprotein. *Anal. Chim. Acta* **893**, 49-56 (2015).
9. Yang, Z. et al. Hollow platinum decorated Fe_3O_4 nanoparticles as peroxidase mimetic couple with glucose oxidase for pseudobienzyme electrochemical immunosensor. *Sensor. Actuat. B-Chem.* **193**, 461-466 (2014).
10. Li, X. et al. Enhanced electrochemiluminescence from luminol at carboxyl graphene for detection of α-fetoprotein. *Anal. Biochem.* **457**, 59-64 (2014).
11. Parshetti, G.K., Lin, F.H. & Doong, R.A. Sensitive amperometric immunosensor for α-fetoprotein detection based on multifunctional dumbbell-like Au-Fe_3O_4 heterostructures. *Sensor. Actuat. B-Chem.* **186**, 34-43 (2013).
12. Zhou, J. et al. A novel electrochemiluminescent immunosensor based on the quenching effect of aminated graphene on nitrogen-doped carbon quantum dots. *Anal. Chim. Acta* **889**, 82-89 (2015).
13. Wang, H. et al. Label-free immunosensor based on Pd nanoplates for amperometric immunoassay of alpha-fetoprotein. *Biosens. Bioelectron.* **53**, 305-309 (2014).
14. Qi, T.T. et al. Label-free alpha fetoprotein immunosensor established by the facile synthesis of a palladium-graphene nanocomposite. *Biosens. Bioelectron.* **61**, 245-250 (2014).
15. Zhao, L. et al. Enzyme-free electrochemical immunosensor configured with Au-Pd

nanocrystals and N-doped graphene sheets for sensitive detection of AFP. *Biosens. Bioelectron.* **49**, 222-225 (2013).

16. Fang, X., Han, M., Lu, G., Tu, W. & Dai, Z. Electrochemiluminescence of CdSe quantum dots for highly sensitive competitive immunosensing. *Sensor. Actuat. B-Chem.* **168**, 271-276 (2012).
17. Xu, R. et al. A sensitive photoelectrochemical biosensor for AFP detection based on ZnO inverse opal electrodes with signal amplification of CdS-QDs. *Biosens. Bioelectron.* **74**, 411-417 (2015).
18. Liang, R.P., Wang, Z.X., Zhang, L. & Qiu, J.D. A label-free amperometric immunosensor for alpha-fetoprotein determination based on highly ordered porous multi-walled carbon nanotubes/silica nanoparticles array platform. *Sensor. Actuat. B-Chem.* **166–167**, 569-575 (2012).
19. Yuan, S. et al. Sandwich-type electrochemiluminescence immunosensor based on Ru-silica@Au composite nanoparticles labeled anti-AFP. *Talanta* **82**, 1468-1471 (2010).
20. Ding, C., Zhao, F., Ren, R. & Lin, J.M. An electrochemical biosensor for α-fetoprotein based on carbon paste electrode constructed of room temperature ionic liquid and gold nanoparticles. *Talanta* **78**, 1148-1154 (2009).
